# Trends in pharmacological prescriptions and polypharmacy for personality disorders: a 10-year cross-sectional analysis of naturalistic data

**DOI:** 10.1186/s12888-025-06716-4

**Published:** 2025-04-01

**Authors:** Flavio Di Leone, Steinn Steingrimsson, Hanne Krage Carlsen, Sophie I. Liljedahl, Peter Sand

**Affiliations:** 1 Department of Psychiatry for Affective Disorders, Region Västra Götaland, Sahlgrenska University Hospital, Gothenburg, Sweden; 2 Section of Psychiatry and Neurochemistry, Institute of Neuroscience and Physiology, Sahlgrenska Academy, University of Gothenburg, Gothenburg, Sweden; 3 Department of Psychology, University of Gothenburg, Gothenburg, Sweden

**Keywords:** Personality Disorders (PD), Pharmacological Prescriptions, Swedish National Guidelines, Polypharmacy, Comorbidity, Register Study, Prescription Trends

## Abstract

**Introduction:**

The purpose of this study was to examine trends in prescribing practices for individuals diagnosed with personality disorders (PD) over a 10-year period in a major metropolitan area in Sweden. Our aim was to assess the alignment of prescribing patterns with national clinical guidelines.

**Methods:**

A register analysis was conducted on 26,520 pharmacological prescriptions from patients' Electronic Health Records (EHRs), documented between January 2011 and December 2020. The study compared the annual proportion of prescriptions across various psychotropic medication classes over time between individuals diagnosed with PD alone and those diagnosed with PD and co-occurring clinical conditions. Additionally, polypharmacy (≥ 3 psychiatric medications) was investigated in both groups.

**Results:**

The proportion of individuals diagnosed with PD alone who received medication increased significantly over the study period. No significant changes were observed in polypharmacy, which remained prevalent in both groups. In the PD alone group, significant negative trends were observed in prescriptions for antidepressants, mood stabilizers, and benzodiazepine derivatives, while stimulant prescriptions rose significantly. In contrast, non-benzodiazepine sedatives and antipsychotics increased significantly in the group with co-occurring conditions.

**Conclusion:**

Our study indicates mixed adherence to Swedish national prescribing guidelines for PD. While there was alignment with recommendations, such as reduced benzodiazepine use, challenges remain in addressing polypharmacy and the prescribing of medications without clear indications, particularly in individuals without comorbidities. These findings emphasize the need for improved diagnostic accuracy, ongoing clinician education, and the integration of prescribing data with diagnostic and treatment records. Future research should explore prescribing practices across various clinical settings and assess the influence of psychopharmacological trends on prescribing, while also defining polypharmacy in the context of personality disorders to guide clinical practice.

## Introduction

Individuals diagnosed with personality disorders (PD) are commonly prescribed psychotropic medications, even though no medications have been approved for these conditions [10]. Meta-analyses provide some support for the use of medication to reduce the intensity of common symptoms of borderline personality disorder (BPD). However, there is little evidence for pharmacotherapy in the treatment of other PDs and their overall efficacy is still unknown [33].

International guidelines advocate a cautious approach to prescribing medications for PD but differ in their aims and modalities of psychopharmacological treatments [30]. Recommendations generated from clinical guidelines are predominantly derived from evidence in relation to BPD and are sometimes extended to PDs as a class. Collectively, guidelines unanimously discourage polypharmacy, defined as the use of multiple medications in combination, but lack clear recommendations on how to avoid this practice [34]. Recommendations do emphasize the importance of communicating the rationale of pharmacological treatments to those receiving treatment, alongside risk and benefit profiles, and thoroughly documenting this process. However, they often lack practical guidance on how medications should be administered or discontinued [20].

The Swedish Psychiatric Association guidelines for PDs, first published in 2008 and amended in 2017, strongly discourage pharmacological treatment for PDs' core symptoms and emphatically discourage polypharmacy [8]. However, these guidelines do encourage the use of psychotropic medications as an addition to other treatments for symptom relief and in cases of severe crisis, while avoiding drugs with addictive potential such as benzodiazepines. Regarding co-occurring clinical conditions, Swedish guidelines state that "*patients should receive pharmacological treatment consistent with the approach used for those without concurrent personality disorders*" [8].

Despite these recommendations, retrospective studies have shown minimal impact on providers’ prescribing patterns and the outcomes for those receiving pharmacotherapy [5, 17, 32]. As a result, virtually all individuals diagnosed with PDs are prescribed at least one psychotropic medication, regardless of the presence of co-occurrent psychiatric conditions [23, 22]. Polypharmacy remains very common and has shown little change over the years [34]. Changes in prescribing practices appear to be mainly due to general trends within the field, such as the introduction of new medications and the discontinuation of those that are outdated [21, 26].

### Aim of the Study

This study aims to analyze pharmacological prescribing patterns for PD over a 10-year period (2011–2020). By examining these trends, we aim to illuminate the real-world pharmacological management of PD and co-occurring psychiatric conditions and to assess alignment with Swedish national guidelines in the context of a register study. These findings may inform efforts to enhance PD treatment strategies, optimize healthcare costs, guide resource allocation within the public healthcare system, and reduce suffering for those receiving treatment.

Based on Swedish national guidelines, the following hypotheses were formulated: (a) psychotropic prescriptions will predominantly target individuals with PD and co-occurring clinical conditions, with this pattern remaining consistent over time; (b) the prescription of benzodiazepines will be infrequent and/or decrease throughout the decade; and (c) the occurrence of polypharmacy—defined as the concomitant use of three or more psychotropic medications—will be rare and mostly confined to individuals with co-occurring psychiatric conditions, with little variation in this trend over time.

## Methods

### Study design

This study employs a 10-year cross-sectional analysis of real-world clinical data to examine trends in pharmacological prescriptions and polypharmacy patterns for personality disorders, focusing on alignment with treatment guidelines.

The study was approved by the Swedish Ethical Review Authority (Dnr 2020–07105). Since the study used administrative pseudonymized administrative register data, the Ethical Review Authority concluded that written consent was not required.

The dataset consisted of prescriptions issued across outpatient and inpatient psychiatric services, excluding the Department of Forensic Psychiatry, at Sahlgrenska University Hospital in Gothenburg, Sweden, between January 2011 and December 2020. Data were extracted from patients' Electronic Health Records (EHRs). The decision to include prescriptions from 2011 onward was based on the implementation of EHRs at the hospital in 2010, ensuring reliable data collection. The observation period ends in 2020, marking the last year before a significant organizational restructuring at the institution that could influence prescription practices. During the intervening decade (2011–2020), the organizational structure remained relatively stable, minimizing external influences on prescription trends.

The inclusion criterion for the register study was that prescriptions were issued for individuals diagnosed with at least one PD diagnostic code based on the *International Statistical Classification of Diseases and Related Health Problems, 10th Revision, Swedish Version* (ICD-10-SE, 2022). Data on co-occurring clinical conditions, age, and sex were then collected.

Prescriptions were excluded if they were intended solely for inpatient use, issued by professional home services, or associated with non-psychiatric conditions (non-F codes according to ICD-10-SE). No additional exclusion criteria were applied.

Prescribed medications were identified using the Anatomical Therapeutic Chemical (ATC) Classification System codes and grouped in therapeutic classes according to the recommendations of the Swedish Psychiatric Association [1], as detailed in Table 1.

**Table 1:** Medications included in the Study, grouped by therapeutic classes, with corresponding Anatomical Therapeutic Chemical (ATC) Classification System codes

Therapeutic Class	Medication Name	ATC Code
Antidepressants (AD)	Agomelatin	N06AX22
	Amitriptyline	N06AA09
	Bupropion	N06AX12
	Citalopram	N06AB04
	Duloxetine	N06AX21
	Clomipramine	N06AA04
	Escitalopram	N06AB10
	Fluoxetine	N06AB03
	Fluvoxamine	N06AB08
	Maprotiline	N06AA21
	Mianserin	N06AX03
	Mirtazapine	N06AX11
	Nortriptyline	N06AA10
	Paroxetine	N06AB05
	Sertraline	N06AB06
	Venlafaxine	N06AX16
**Antipsychotics (AP)**	Aripiprazole	N05AX12
	Fluphenazine	N05AB02
	Flupentixol	N05AF01
	Haloperidol	N05AD01
	Clozapine	N05AH02
	Quetiapine	N05AH04
	Risperidone	N05AX08
	Levomepromazine	N05AA02
	Lurasidone	N05AX13
	Melperon	N05AD03
	Olanzapine	N05AH03
	Paliperidone	N05AX13
	Perphenazine	N05AB03
	Ziprasidone	N05AE04
	Zuclopenthixol	N05AF05
**Mood Stabilizers (MS)**	Gabapentin	N03AX12
	Carbamazepine	N03AF01
	Lamotrigine	N03AX09
	Oxcarbazepine	N03AF02
	Pregabalin	N03AX16
	Topiramate	N03AX11
	Valproic Acid	N03AG01
**Stimulants (CS)**	Amphetamine	N06BA01
	Atomoxetine	N06BA09
	Dexamfetamine	N06BA02
	Lisdexamfetamine	N06BA12
	Methylphenidate	N06BA04
	Modafinil	N06BA07
**Benzodiazepines (BZ)**	Alprazolam	N05BA12
	Diazepam	N05BA01
	Flunitrazepam	N05CD03
	Clonazepam	N03AE01
	Lorazepam	N05BA06
	Nitrazepam	N05CD02
	Midazolam	N05CD08
	Oxazepam	N05BA04
	Triazolam	N05CD05
**Benzodiazepine Derivatives (Z)**	Zaleplon	N05CF03
	Zolpidem	N05CF02
	Zopiclone	N05CF01
	Clobazam	N05BA09
**Non-Benzodiazepine Sedatives and Anxiolytics (NBSA)**	Alimemazine	R06AD01
	Buspirone	N05BE01
	Hydroxyzine	N05BB01
	Promethazine	R06AD02
	Propiomazine	N05CM06
	Melatonin	N05CH01

Medications commonly prescribed off-label for psychiatric symptoms, such as propranolol and prazosin — for anxiety and for trauma-related nightmares respectively — were excluded from the current study, due to the inability to reliably differentiate between prescriptions for psychiatric versus somatic indications within the available data.

To assess polypharmacy, a threshold was defined as the simultaneous use of three or more psychotropic medications. This numerical conceptualization is reported in several studies that have examined prescribing patterns in the same population, such as those by Pascual et al. [22], Soler et al. [31], and Tennant et al. [34]. However, it is important to note that definitions of polypharmacy are not standardized, with other studies using a threshold of five or more medications in their analysis of older population diagnosed with PD [28]. We decided to adopt a threshold of three medications because the study population was moderately young (mean age 35.07 ± 11.1 years), and the dataset exclusively included psychotropic prescriptions, excluding medications intended for somatic use. This approach ensures a clinically relevant definition of polypharmacy for this demographic and facilitates comparisons with studies using similar thresholds.

### Statistical analysis

The initial dataset consisted of 26,520 prescriptions, issued for 4,461 individuals diagnosed with PD at the time of the record. Each individual received between 1 and 74 prescriptions per year (mean: 5.5; median: 6.6), with many contributing prescription data over multiple years. To focus on unique pharmacological prescriptions, records of the same medication prescribed to the same individual within a single year were excluded, reducing the dataset to 10,295 unique prescription records.

Data were categorized into two groups based on prescriptions for individuals diagnosed with PD and co-occurring clinical conditions (PD with comorbidities), and prescriptions for individuals diagnosed exclusively with PD (PD alone).

To test the formulated hypotheses, the analyses focused on the following outcomes:

1. Time trends in proportion of prescriptions for the PD with comorbidities group compared to the prescriptions in the PD alone group.
2. Time trends in the proportion of prescriptions for each therapeutic classes (Table 1) for both groups (PD alone and PD with comorbidities).
3. Time trends in the proportion of individuals receiving more than three prescriptions (polypharmacy) in both groups (PD alone and PD with comorbidities).

Outcomes for diagnostic and prescription patterns were presented graphically, accompanied by *p*-values derived from correlation analysis (Pearson’s R).

Results are reported as correlation coefficients between year and polypharmacy percentage, along with *p*-values for these correlations. Statistical significance was defined as a *p*-value less than 0.05. To account for multiple outcomes, *p*-values were corrected using the Bonferroni correction to ensure the robustness of statistical significance.

## Results

### Changes in population demographics

The total number of individuals prescribed psychotropic medications for PD increased fourfold over the observation period, aligning with the rise in the number of individuals receiving psychiatric care at Sahlgrenska University Hospital (Table 2).

**Table 2:** Descriptive Statistics of the Study Population by Year: Age, Gender, and Comorbid Diagnostic Classes with respective ICD-10 Codes

	Individuals receiving medical prescriptions per year	Age		Females		Disorders of personality Without comorbidity (F600-F629)		Hyperkinetic disorders (F900-F909)		Neurotic, stress-related and somatoform disorders (F40-F48)		Autism spectrum disorders (F840-F849)		Intellectual Disabilities (F70-F79)		Bipolar Mood Disorders (F310-319)		Unipolar mood disorders (F320-F349)		Schizophrenia, schizotypal and delusional disorders (F20-F29)		Mental and behavioural disorders due to psychoactive substance use (F10-F19)	
year	n	mean	SD	n	%	n	%	n	%	n	%	n	%	n	%	n	%	n	%	n	%	n	%
2011	396	34.38	10.3	277	69.95	81	20.45	37	9.34	189	47.73	6	1.52	4	1.01	40	10.1	113	28.54	15	3.79	11	2.78
2012	666	34.44	10.8	487	73.12	151	22.67	78	11.71	251	37.69	9	1.35	4	0.6	73	10.96	177	26.58	20	3	54	8.11
2013	892	35.27	11.3	623	69.84	184	20.63	132	14.8	310	34.75	16	1.79	7	0.78	95	10.65	228	25.56	28	3.14	119	13.34
2014	945	35.21	10.96	687	72.7	190	20.11	156	16.51	350	37.04	28	2.96	10	1.06	96	10.16	233	24.66	30	3.17	127	13.44
2015	1057	35.1	11.04	785	74.27	255	24.12	194	18.35	386	36.52	33	3.12	12	1.14	90	8.51	231	21.85	28	2.65	106	10.03
2016	1201	34.8	10.93	890	74.1	284	23.65	230	19.15	449	37.39	47	3.91	11	0.92	105	8.74	245	20.4	41	3.41	113	9.41
2017	1301	35.04	10.8	981	75.4	322	24.75	244	18.75	481	36.97	47	3.61	11	0.85	113	8.69	263	20.22	46	3.54	108	8.3
2018	1307	35.2	10.94	993	75.98	344	26.32	239	18.29	465	35.58	39	2.98	12	0.92	100	7.65	230	17.6	45	3.44	97	7.42
2019	1302	35.26	10.97	1007	77.34	325	24.96	246	18.89	483	37.1	42	3.23	14	1.08	100	7.68	242	18.59	38	2.92	99	7.6
2020	1228	35.72	10.84	939	76.47	303	24.67	236	19.22	456	37.13	44	3.58	15	1.22	89	7.25	221	18	31	2.52	98	7.98

The mean age remained relatively unchanged during the observation period, reflecting a turnover in the patient group, while the standard deviation remained stable, indicating a consistent level of variability within the age distribution over the years.

The gender distribution among individuals with PD alone remained stable over the study period, with females comprising approximately 75% of the sample. In contrast, the proportion of females in the group with co-occurring clinical conditions showed a slight decline over time. For neurodevelopmental conditions, the gender distribution remained relatively balanced, with females accounting for 69% to 77%. However, in individuals with bipolar and unipolar disorders, the proportion of females was significantly lower, ranging from 17 to 26%.

### Changes in diagnostic patterns

Notably, the proportion of individuals diagnosed with PD without co-occurring clinical conditions increased significantly over time (R = 0.81, *p* = 0.0045).

**Fig. 1:**
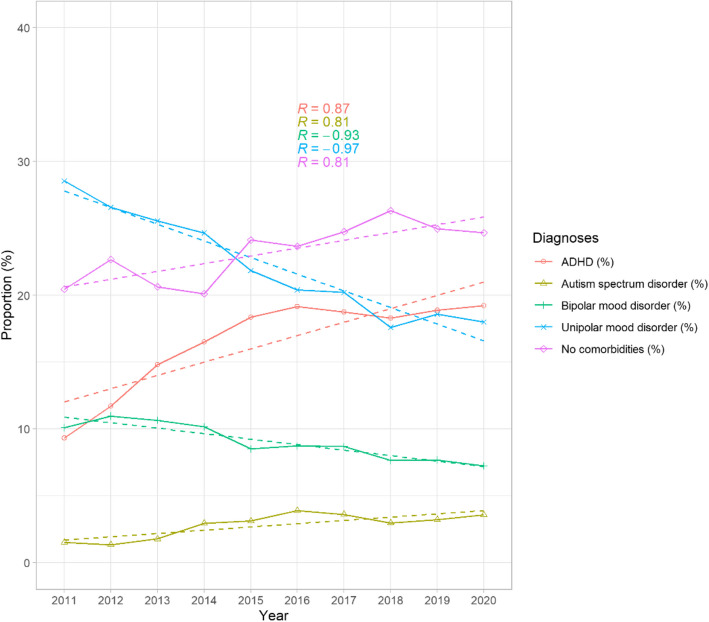
Proportion (%) of individuals with personality disorders (PD) with and without other comorbid diagnoses by year. Significant increases were observed in co-occurring attention-deficit/hyperactivity disorder (ADHD, *p* = 0.006) and autism spectrum disorders (ASD, *p* = 0.028), while the proportions of comorbid bipolar disorder and unipolar depression declined notably (both *p* < 0.001). The proportion of individuals diagnosed with personality disorders (PD) alone also increased significantly over time (*p* = 0.0045). No significant trends were observed for psychosis (*p* = 1), intellectual disability (*p* = 0.66), substance use disorders (*p* = 1), or anxiety disorders (*p* = 1), which are not shown in the figure

Regarding diagnostic patterns, the proportion of individuals with co-occurring attention-deficit/hyperactivity disorder (ADHD) and autism spectrum disorders (ASD) showed a consistent upward trend throughout the study period, increasing from 9 to 19% (Fig. 1, R = 0.87, *p* = 0.006) and from 1.5 to 3.6% (R = 0.81, *p* = 0.028), respectively. Conversely, there was a notable decline in the proportion of participants diagnosed with concomitant bipolar and unipolar depression (R = −0.93, *p* < 0.001 and R-0.97, *p* < 0.001 respectively). The proportion of individuals diagnosed with co-occurring intellectual disability, psychosis, substance use disorders (SUD), and anxiety disorders did not show significant associations with time during the study period.

### Trends in pharmacological prescriptions

**Fig. 2:**
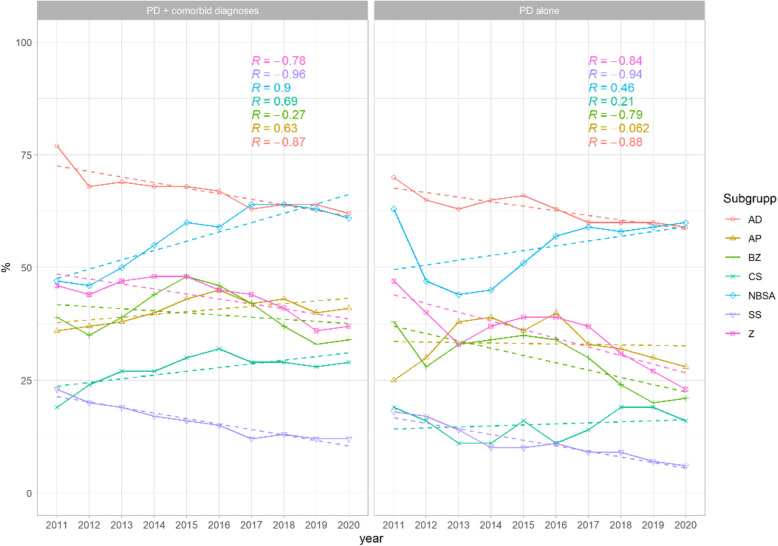
Proportion of prescribed medications by therapeutic classes by year in individuals with personality disorders (PD) and other comorbid diagnoses (left) and PD alone (right). The figure shows the distribution of prescribed medications across different therapeutic classes for individuals with Personality Disorders (PD) and additional comorbid diagnoses (left) and for those with PD alone (right). Significant decreases in prescriptions were observed for antidepressants (AD, *p* < 0.001 for PD alone, *p* < 0.05 for PD with co-occurring psychiatric conditions) and mood stabilizers (MS, *p* = 0.0049 for PD alone, *p* < 0.001 for PD with co-occurring psychiatric conditions). Non-benzodiazepine sedatives and anxiolytics (NBSA) showed a significant increase in the group with co-occurring psychiatric conditions (*p* < 0.001). Antipsychotic (AP) prescriptions also increased significantly for this group (*p* = 0.01). No significant trends were found for benzodiazepine derivatives (Z, *p* = 0.35 for PD alone, *p* = 0.6 for PD with co-occurring psychiatric conditions), benzodiazepine prescriptions (BZ, *p* = 0.007 for PD alone, p = 1 for PD with cooccurring psychiatric conditions), or stimulant (CS) prescriptions in the group with co-occurring psychiatric conditions (*p* = 1). However, stimulant prescriptions increased significantly in the PD-alone group (*p* < 0.05)

Changes in pharmacological prescription patterns were observed in both groups (Fig. 2). Significant negative time trends were found for antidepressants (AD), with a strong negative correlation for the PD-alone group (R = −0.84, *p* < 0.001). The trend remained negative in the group with co-occurring psychiatric conditions, though less pronounced (R = −0.78, *p* < 0.05). The proportion of prescriptions for mood stabilizers (MS) showed a significant decrease in prescriptions for both groups (PD alone: R = −0.88, p = 0.0049; PD with co-occurring clinical conditions: R = −0.87, *p* < 0.001).

A negative trend was noted also for benzodiazepine derivatives (Z), although neither group showed a statistically significant change (PD alone: R = −0.63, p = 0.35; PD with co-occurring psychiatric conditions: R = −0.062, *p* = 0.6). Benzodiazepine prescriptions (BZ) showed a negative time trend only in the PD-alone group (R = −0.46, *p* < 0.007), with no significant change in the group with co-occurring clinical conditions (R = −0.27, *p* = 1).

In contrast, the proportion of prescriptions for non-benzodiazepine sedatives and anxiolytics (NBSA) increased significantly in the group with co-occurring clinical conditions (R = 0.9, *p* < 0.001), while the trend in the PD-alone group remained constant (R = 0.46, *p* = 1). The proportion of stimulant prescriptions (CS) increased in the PD-alone group (R = 0.69, *p* < 0.05), but the trend was not significant for those with co-occurring clinical conditions (R = 0.21, *p* = 1). Finally, the proportion of prescriptions for antipsychotics (AP) showed a non-significant negative trend in the PD-alone group (R = −0.062, *p* = 0.06), while the group with co-occurring clinical conditions exhibited a significant increase (R = 0.63, *p* = 0.01).

### Polypharmacy

**Fig.3:**
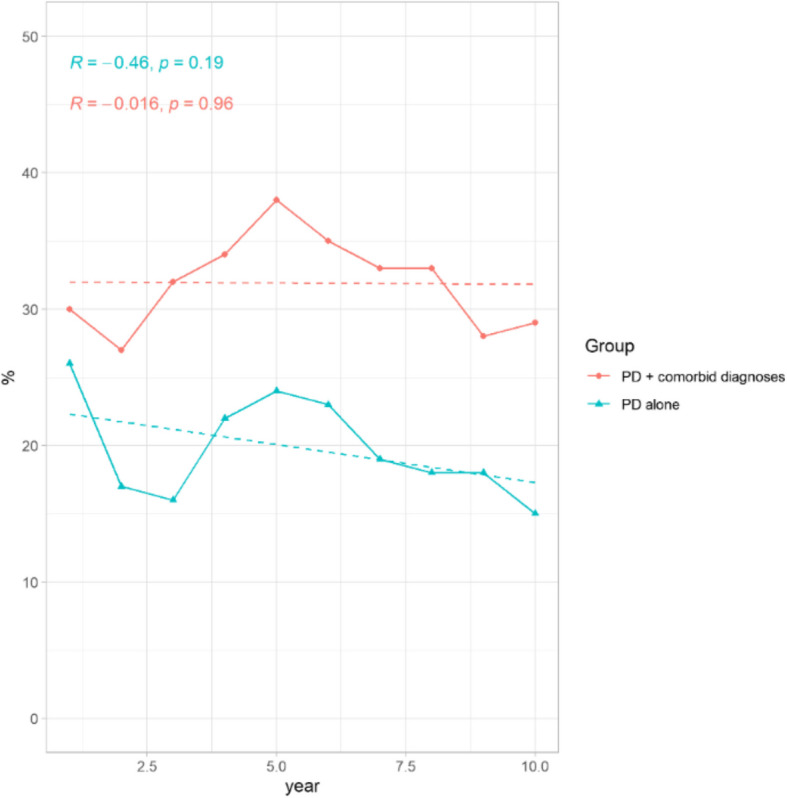
Proportion (%) of the study population with polypharmacy (defined as the concurrent use of ≥ 3 psychiatric medications) by year. No significant trends were found neither in individual with personality disorders (PD) nor PD with co-occurring clinical conditions (*p* = 1 and *p* = 0.38, respectively)

The analysis showed a negative but not significant change in the proportion of individuals with polypharmacy (defined as the concurrent use of ≥ 3 psychiatric medications) during the study period (Fig. 3).

In 2011, polypharmacy, defined here as the concurrent use of three or more psychotropic medications, was documented in slightly more than a quarter of individuals diagnosed with PD alone and in one-third of those with co-occurring psychiatric conditions. By 2020, the prevalence of polypharmacy among individuals with co-occurring conditions remained stable (R = −0.016, *p* = 1). Meanwhile, the frequency of polypharmacy in the PD-only group decreased to 15%, although this trend was not statistically significant (R = −0.46, *p* = 0.38).

### Discussion

Our main findings highlight that alignment with Swedish national guidelines for benzodiazepine use improved significantly, with a notable reduction in prescriptions over the study period. However, polypharmacy remained prevalent, especially among individuals without documented co-occurring clinical conditions. Further, the practice of prescribing of pharmacological treatments to individuals with PD alone increased over time, contrary to recommendations generated from national guidelines.

Other naturalistic studies across various countries have observed similar trends [29]. Pascual et al.'s studies from 2010 to 2021 found that individuals diagnosed with BPD are often prescribed off-label medications, with a significant decrease in benzodiazepine use and an increase in prescriptions for second-generation antipsychotics. These findings are primarily attributed to general changes in psychiatric practice over time, rather than specific interventions for personality disorders (PD). Analyses from Italy and the United Kingdom have reported similar findings [7, 19]. These studies collectively highlight the evolving landscape of psychotropic pharmacological interventions for PD, reflecting ongoing efforts in clinical practice to optimize treatment by reducing the use of drugs considered dangerous, with addictive potential, or ineffective. However, despite these efforts, the goal of minimizing or containing pharmacological treatment for PD, as recommended by guidelines, has not been fully achieved [6].

### Hypothesis (a): Pharmacological prescriptions would almost exclusively pertain to individuals with co-occurring clinical conditions.

Contrary to this hypothesis and national guidelines, which generally discourage pharmacotherapy in absence of co-occurring clinical conditions, the proportion of individuals diagnosed with PD alone who were treated with medication has significantly increased over the study period. Several potential explanations for this increase could be considered.

One possible explanation is that physicians may simply disregard the guidelines. A recent qualitative study explored psychiatrists' perspectives on prescribing for individuals diagnosed with PD and found that the European guidelines, including those in Sweden, are viewed as ambiguous and lacking clear structure [27]. It appears that clinicians rely on their own experience or that of their colleagues when selecting and administering pharmacological treatments. This could also open the door to broader pharmacological trends, such as the release of new medications or treatment modalities, which may further influence prescribing practices outside the scope of established guidelines.

Another possibility is the adoption of symptom-based or trait-based pharmacological strategies for treating PD. For example, while European guidelines generally advise against pharmacotherapy for PD, the American Psychiatric Association’s 2001 practice guideline for borderline personality disorder (BPD), which was in use during the study observation period, recommends pharmacotherapy to address specific core symptoms such as affective instability, impulsivity, psychotic-like symptoms, and self-destructive behavior [3]. The updated APA guideline [2] maintains a symptom-oriented approach but adopts a stance more in line with the restrictive European guidelines. It recommends pharmacotherapy as a time-limited intervention and strictly as an adjunct to psychotherapy, which remains the cornerstone of treatment. The guideline places strong emphasis on clinical utility, highlighting the importance of regular pharmacological reviews and thorough clinical assessments before initiating any new medication.

Symptom-based or trait-based pharmacological strategies has also been incorporated into general psychiatric management strategies for PDs [4, 12]. Adherence to APA guidelines has been associated with an increased reliance on medication for treating of BPD, leading to higher prescription rates, particularly of antidepressants and neuroleptics [15].

Reliance on APA´s 2001 guidelines, rather than European ones, may explain the observed increase in prescription rates within the group without co-occurring clinical conditions in our study. However, the prescription rates for specific therapeutic classes in our findings differ from those reported by Kolla et al. Moreover, since symptom-based or trait-based strategies emphasize pharmacological treatment for managing symptoms, it is theoretically possible that these approaches might lead physicians to overlook or underreport co-occurring affective disorders. The latter scenario may explain the observed decrease in diagnoses of bipolar and unipolar depression in our study, in contrast to the stable rates of co-occurring psychosis, intellectual disabilities, substance use disorder (SUD), and anxiety disorders. The increasing estimates of diagnoses for ADHD and autism spectrum disorders, on the other hand, are consistent with broader epidemiological trends and changes in diagnostic patterns [18, 25].

A final possible interpretation of our findings is that current guideline recommendations, potentially combined with symptom-based strategies, may place too much emphasis on pharmacological treatments when co-occurring clinical symptoms are present. A recent Cochrane review reported small effects for treatment with antipsychotics and anticonvulsants, and no robust support for antidepressants, on co-occurring psychopathology in individuals with BPD [24]. These findings cast doubt on the validity of guideline recommendations to treat comorbidity in a manner "consistent with the approach used for those without concurrent personality disorders" ([8], p. 136). Such strategies may overlook the complexities of co-occurring psychiatric conditions in PD: whether they represent distinct disorders—one treatable with medication and the other, PD, not—or interconnected issues requiring a more integrated treatment approach beyond combined or adjunct therapies [35]. In the absence of clear diagnostic boundaries, clinicians may apply guideline recommendations to treat depression, psychotic episodes or mood disturbances in PD without fully assessing or documenting them as co-occurrent clinical conditions.

### Hypothesis (b): Prescription of benzodiazepines would be infrequent.

We observed a significant negative time trend for benzodiazepine (BZ) prescriptions among individuals with PD alone, which partially supports our hypothesis. The decrease was, however, less pronounced in the group with co-occurring psychiatric conditions, suggesting that benzodiazepines may still be considered necessary for addressing symptomatology that is difficult to manage with alternative treatments [11]. Moreover, the proportion of benzodiazepine derivative (Z) prescriptions showed no significant change in either group, which may suggest that the total prescriptions for benzodiazepines and their derivatives remain relatively common.

This trend aligns with broader evidence from Højlund et al. [13], which reported a decrease in benzodiazepine prescriptions across all age groups in Sweden from 2004/2006 to 2020, while the use of benzodiazepine derivatives remained stable. The overall decline in benzodiazepine prescriptions in our study may therefore indicate changes in prescribing practices that are not solely influenced by clinical guidelines.

It is crucial to limit the use of potentially harmful medications, but the complexity of prescribing practices for individuals with co-occurring PD suggests that completely avoiding benzodiazepines may not always be practical. First, benzodiazepines may also serve secondary purposes beyond treating PD symptoms or co-occurrent psychiatric conditions, such as managing side effects from other psychiatric medications [9]. Further, although benzodiazepines and their derivatives are generally discouraged due to their association with increased impulsivity and self-destructive behavior—evidence primarily derived from studies on BPD—their actual impact on clinical outcomes in PD pathology remains unclear [14]. Consequently, reducing benzodiazepine prescriptions in this population could have uncertain effects on clinical outcomes (Paton, 2002). Finally, the concurrent rise in prescriptions for antipsychotics (AP) and non-benzodiazepine sedatives and anxiolytics (NBSAs), particularly among individuals with co-occurring clinical syndromes, may suggest a shift toward these medications as alternatives to benzodiazepines, potentially posing risks related to less studied drug interactions and adverse effects that are not fully understood or adequately monitored.

### Hypothesis (c): Polypharmacy would be infrequent and restricted to individuals diagnosed with co-occurring clinical syndromes.

The findings in our study partially support this hypothesis. While there was a noticeable reduction in the proportion of individuals with PD alone receiving pharmacological treatment with more than three medications, polypharmacy remained prevalent, particularly among those with co-occurring clinical conditions. These results are consistent with findings by Pascual et al. [22], who observed a strong correlation between the presence of co-occurring psychopathology and higher rates of polypharmacy in individuals diagnosed with PD. However, our study also revealed a substantial prevalence of polypharmacy among individuals with PD alone, a result that diverges from the expectation that comorbidity is the primary driver of multiple prescriptions.

Given the lack of specific pharmacological indications for PD in current guidelines, even a single prescribed medication could arguably be viewed as overprescribing. The cut-off of three medications used in this study as an indicator of polypharmacy, while widely used in the literature, is inherently arbitrary, as demonstrated by Masnoon et al. [16], who identified more than 138 definitions of polypharmacy and associated terms. This variability underscores the conceptual challenges in defining polypharmacy and evaluating its clinical implications, particularly in the context of PD.

Nevertheless, the consequences of polypharmacy are considerable, especially given the unique risks of overmedication in individuals diagnosed with PD. These risks include heightened likelihood of fatal intoxication from drug interactions, difficulties in deprescribing when needed, and challenges in monitoring the effectiveness and side effects of medications due to complex interactions. These factors underscore the importance of critically evaluating polypharmacy in PD management, ensuring that any treatment plan is carefully tailored to the individual's needs while remaining mindful of the potential harms associated with overmedication.

### Conclusions and future directions

Our research underscores the value of using common population data and real-world practice settings in pharmacoepidemiology, now made more feasible by Electronic Health Records (EHRs).

To achieve a more nuanced understanding of pharmacological management in PD, future research should leverage data sources that detail prescription indications or integrate prescribing data with diagnostic records and treatment goals. This would necessitate distinguishing between primary uses of medications for managing PD symptoms, comorbidity and/or secondary uses. Similarly, studies conducted in other clinical settings, such as primary care, inpatient care, and home services, are crucial as they provide a broader perspective on prescribing practices and may reveal different prescription patterns influenced by the specific context of care.

Changes in prescribing practices can significantly impact resource allocation, budgeting, and cost-effectiveness analyses, highlighting the need for strategic adaptation to evolving clinical trends. It appears that some doctors issuing the prescriptions collected in our study do not consistently categorize medications by pharmacological classes or diagnostic categories [27]. This suggests that prescribing practices may be more influenced by individual clinical judgment and general pharmacological trends rather than adherence to structured treatment protocols. Future research should address the influence of these external factors, which are often poorly studied and understood, yet may drive shifts in prescribing practices, particularly for off-label uses.

Additionally, studies assessing qualitative outcomes of different guideline approaches and recommendation strategies are needed. The relative effectiveness of the North American symptom-oriented approach versus the diagnostic-focused approach of the European guidelines remains an open question. Further exploration is required to determine the circumstances under which each approach confers costs and benefits for individuals receiving treatment.

Finally, our study underscores the urgent need for a clear and standardized definition of polypharmacy in the context of personality disorders (PD) to guide clinical practice and drive interventions where necessary. The high rates of polypharmacy observed in our data reflects the dual challenges clinicians face: addressing severe symptomatology in the absence of evidence-based pharmacological treatments and balancing individualized care with adherence to clinical guidelines. Establishing a consistent definition of polypharmacy is essential for identifying inappropriate prescribing patterns, assessing associated risks, and implementing targeted interventions.

### Strengths and limitations

The retrospective and observational nature of the study precludes the establishment of causal relationships between the observed prescription patterns and specific clinical practices, or guideline changes implemented during the study period. Furthermore, the study's reliance on electronic health record data including incomplete information due to changes in electronic record systems could potentially affect the comprehensiveness and precision of the findings.

The exclusion of inpatient prescriptions and those administered through home healthcare services may result in an underestimation of the full pharmacological treatment patterns across diverse care settings. Additionally, the omission of somatic medications prescribed for psychiatric symptoms, (for example, propranolol and prazosin), potentially underestimates the prevalence of different therapeutic strategies employed in clinical practice.

A significant strength of the present study is the large, naturalistic sample of pharmacological prescriptions for individuals with a registered PD diagnosis. Since guidelines are meant for clinicians to follow, such an unselected sample allows for a reliable generalization of prescription trends over time.

## Data Availability

No datasets were generated or analysed during the current study.
